# Sperm-oocyte interplay: an overview of spermatozoon’s role in oocyte activation and current perspectives in diagnosis and fertility treatment

**DOI:** 10.1186/s13578-020-00520-1

**Published:** 2021-01-06

**Authors:** Mohammad Ishraq Zafar, Shi Lu, Honggang Li

**Affiliations:** 1 Institute of Reproductive Health/Center of Reproductive Medicine, Tongji Medical College, Huazhong University of Science and Technology, 13 Hang Kong Road, Wuhan, 430030 People’s Republic of China; 2 Department of Obstetrics and Gynecology, Union Hospital, Tongji Medical College, Huazhong University of Science and Technology, 1277 Jeifang Avenue, Wuhan, 430022 People’s Republic of China; 3 Wuhan Tongji Reproductive Medicine Hospital, 128 Sanyang Road, Wuhan, 430013 People’s Republic of China

**Keywords:** Oocyte activation, Calcium oscillations, Sperm oocyte activation factors (SOAF), Male infertility diagnosis, Artificial oocyte activators (AOAs), Male infertility, Male infertility treatment

## Abstract

The fertilizing spermatozoon is a highly specialized cell that selects from millions along the female tract until the oocyte. The paternal components influence the oocyte activation during fertilization and are fundamental for normal embryo development; however, the sperm-oocyte interplay is in a continuous debate. This review aims to analyze the available scientific information related to the role of the male gamete in the oocyte activation during fertilization, the process of the interaction of sperm factors with oocyte machinery, and the implications of any alterations in this interplay, as well as the advances and limitations of the reproductive techniques and diagnostic tests. At present, both PLCζ and PAWP are the main candidates as oocyte activated factors during fertilization. While PLCζ mechanism is via IP_3_, how PAWP activates the oocyte still no clear, and these findings are important to study and treat fertilization failure due to oocyte activation, especially when one of the causes is the deficiency of PLCζ in the sperm. However, no diagnostic test has been developed to establish the amount of PLCζ, the protocol to treat this type of pathologies is broad, including treatment with ionophores, sperm selection improvement, and microinjection with PLCζ protein or RNA.

## Background

In animals, gamete identification and other findings showing that the spermatozoon enters the oocyte to form the embryo, leading to new hypotheses and experiments for discovering the mechanisms of the fertilization process.

In mammals, fertilization involves a series of consecutive steps that starts with the recognition and fusion of sperm and oocyte membranes. This event triggers a pathway that induces persistent cytosolic calcium (Ca^+ 2^) oscillations, which are necessary and sufficient to stimulate embryo development [1]. The Ca^+ 2^ oscillations last for several hours [2, 3], are the common signal of oocyte activation, and start the intricate embryonic development process to form a zygote.

It was believed that the spermatozoon’s only contribution to the embryo formation was its genome. In recent times, extensive studies have shown that the spermatozoon contribution is substantial; it contributes both its DNA and its entire structure to embryo formation [4]. Upon fertilization, sperm-specific proteins and factors trigger Ca^+ 2^ oscillations to activate the oocyte. While the sperm centriole guides both oocyte and sperm nuclei to form the zygote nucleus and sperm DNA structures, chromatin and free RNAs can be modified to activate/deactivate gene expression involved in embryo development [5]. These interactions demonstrate that the spermatozoon has an active role in both oocyte activation and zygote formation, affecting the embryo’s phenotype directly.

Since 2002, numerous studies have shown that the sperm oocyte-activator factor (SOAF), phospholipase C zeta (PLCζ), is involved in oocyte activation, promoting MII resumption and pronuclear formation through the inositol-1,4,5-triphosphate (IP_3_) pathway. In 2007 the PAWP protein was proposed to be a SOAF [6]. The role and importance of each protein, and the pathway for oocyte activation, are new topics of debate among investigators worldwide. One goal is to identify specific receptors within the oocyte that interact with these factors, triggering Ca^+ 2^ oscillations and oocyte activation upon fertilization.

Advances in reproductive biotechnological medicine have helped to clarify our understanding of the stages of fertilization and embryo development. At present, intracytoplasmic sperm injection (ICSI) is widely used in assisted reproductive technology (ART). The aim of ART is to achieve fertilization by directly injecting the sperm into the oocyte by passing the many biological barriers in the process [7]. Continuous improvement in ART has allowed severe infertility cases to be successful, even when recurrent fertilization failures occur after conventional in vitro fertilization (IVF).

The total fertilization failure (TFF) is when all the oocytes collected within one cycle of stimulation fail to form pronuclei, with oocyte activation deficiency (OAD) as the primary cause of such failures [8]. It has been demonstrated that PLCζ protein absence in the sperm head is associated with direct failure to signal Ca^+ 2^ oscillations [8]. While ICSI has a high rate of success, there are still cases of OAD involving ICSI failure. Pharmaceutical alternatives to activate the oocyte artificially are Ca^+ 2^ ionophores, known as artificial oocyte activators (AOAs). Modifications of reproductive technologies such as intracytoplasmic sperm injection followed by microinjections of mRNA PLCζ and recombinant active PLCζ protein [9] have been developed for cases where AOAs have failed. Therefore, it has been suggested that PLCζ could be a fundamental clinical diagnosis biomarker [10]. However, there is a need to establish diagnostic protocols and reference clinical ranges to apply this treatment to infertile patients.

The aim of this review is to select and discuss reports that elucidate the importance of the spermatozoon in fertilization and its active role in mammalian oocyte activation. We intend to clarify the complex mechanism of oocyte activation and propose new ART strategies that can be applied in human reproductive pathologies.

## From membrane fusion to oocyte activation

**Fig. 1:**
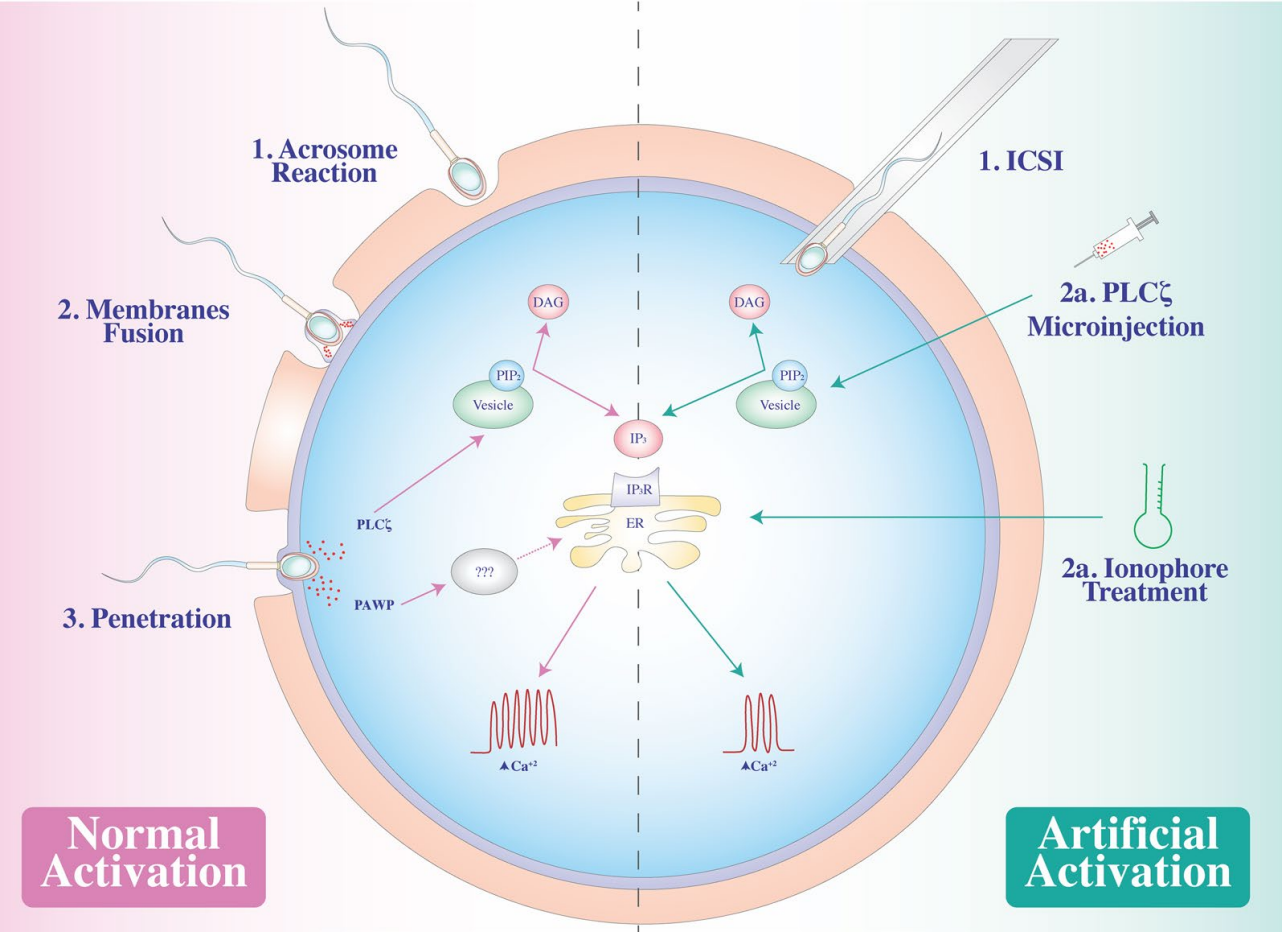
The role of the sperm factors in the oocyte activation. In the *normal activation* process, the acrosome reaction (1) allows the exposure of PT for the fusion of the sperm membrane with the oocyte (2), starting in the equatorial segment and continuing to the PAS-PT, until penetration (3). SOAF are released into the ooplasm and triggers the Ca^+ 2^ oscillations via IP_3_. In the *artificial oocyte activation*, different strategies following ICSI could trigger Ca^+ 2^ oscillations, PLCζ microinjections (2a) or ionophore treatments (2b)

Upon fertilization, a signaling pathway induces the cortical granules in the ooplasm (the oocyte cytoplasm) to fuse with the oocyte membrane and release their contents into the extracellular matrix (Fig. 1—normal activation). This phenomenon, known as the cortical reaction, is associated with another event called the zona reaction, which is the modification of the structure of the zona pellucida to block polyspermy and protect the developing embryo during implantation. What triggers this sequence of events remains unknown.

Since the establishment of Loeb´s principle, which states that the spermatozoon has major roles in fertilization, promotion of cell division and paternal inherence to the offspring, a series of studies and discussions have taken place within the scientific community [11]. At present, the second role of the spermatozoon, as per Loeb, is fundamental to initiating oocyte activation, a complex series of events that involve both the sperm and oocyte factors.

Once the oocyte recognizes the spermatozoa, the sperm binds the zona pellucida protein ZP-3, which functions as a sperm recognition receptor. The acrosomal membrane surrounding the spermatozoon head (Figs. 1—normal activation and  2) reacts and fuses with the oocyte membrane in a phenomenon called the acrosome reaction. A sperm membrane protein, Izumo1, binds to its counterpart Juno, an oocyte receptor, and trigger the membrane fusion of the two gametes. This interaction has been identified as essential and extremely regulated and is the culmination of the fertilization process [12]. The fusion of the gametes’ membrane allows sperm factor entry. These events trigger a signaling pathway in the ooplasm that releases intracellular calcium (Ca^+ 2^), leading to the exocytosis of cortical granules to the extracellular matrix followed by pronuclei formation, maternal mRNA recruitment, and release from meiotic arrest. This series of events leads to forming a single activated cell, the zygote, and initiation of embryonic gene expression.

**Fig. 2:**
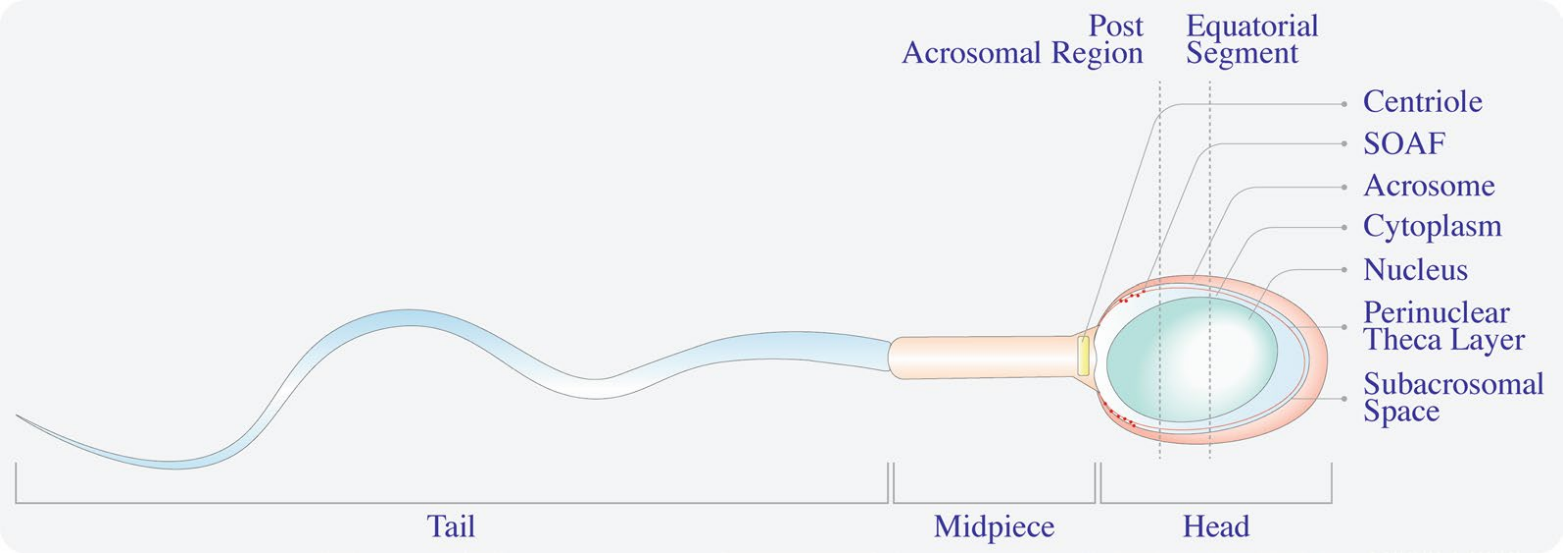
Spermatozoon acrosomal organization. The spermatozoon has three fundamental pieces: tail, middle, and head. The sperm head has a prominent and compact nucleus, surrounded by a thin cytoplasm that contains nuclear vesicles. The PT is a thin protein layer underneath the acrosomal membrane and is divided into a subacrosomal region, equatorial segment, and PAS-PT. The PAS-PT contains SOAF and is the first to be exposed in the ooplasm during fertilization

In mammals, fluorescent Ca^+ 2^ sensitive dye techniques help confirm that sperm entry causes persistent oscillations of intracellular Ca^+ 2^ in the ooplasm [13]. The release of Ca^+ 2^ is the common signal of oocyte activation [14].

## Ca^+ 2^ signaling

Since the 1970s, it has been believed that an increase in the levels of intracellular Ca^+ 2^ in the ooplasm during fertilization is a fundamental signal for the fertilization process to succeed. Studies in mammals have shown that intracellular Ca^+ 2^ is stored in the endoplasmic reticulum (ER) and mobilizes to the ooplasm in spatial-temporal waves. Ca^+ 2^-sensitive fluorescent dye techniques have established Ca^+ 2^ and oscillation patterns in mammals, including pigs, rats, cows, mice, and humans. The frequency and amplitude of Ca^+ 2^ release patterns are crucial for oocyte activation and the preliminary stages of embryogenesis to occur, thereby are distinctive of each species. For example, a low frequency with one Ca^+ 2^ spike occurring every 10 minutes was observed in mouse oocytes, while in humans, pigs, and cows, each Ca^+ 2^ spike occurred once every 30 to 60 min [13, 15].

In mammalian fertilization, the rise of intracellular Ca^+ 2^ depends on the activation of inositol-1,4,5-triphosphate receptors (IP_3_R) located in specialized compartments of the ER membrane [14, 16, 17] (Fig. 1—normal activation). This process is known as Ca^+ 2^-induced Ca^+ 2^ release (CICR process) and is based on two types of Ca^+ 2^ channels: IP_3_R and ryanodine receptors (RYR). Both types of channels are Ca^+ 2^-depending for stimulation and inhibition, so during the process, these channels open and close in order the discharged Ca^+ 2^ from the internal ER store, the rise of cytoplasmic Ca^+ 2^ inactivates the receptors, and the Ca^+ 2^ is driven back to the store, the cytoplasmic concentration returns to the basal status, and the cycles starts again. Although this mechanism is not fully understood, it is known that the penetration of the spermatozoon triggers the CRIC process [18].

Regulating these IP_3_ signaling pathways in the oocyte is phospholipase C (PLC). This cytosolic enzyme catalyzes the hydrolysis of phosphatidylinositol 4,5-biphosphate (PIP_2_) in IP_3_ and diacylglycerol (DAG). Store-operated calcium entry, involving a group of molecules including STIMI, ORAII, and SERCA, plays a key role in Ca^+ 2^ homeostasis. This mechanism allows the refill of the ER with free Ca^+ 2^ and generation of Ca^+ 2^ oscillations that are observed in oocyte activation [19]. Although the fully Ca^+ 2^ oscillations mechanism, and its relationship with the completion of meiosis, are unknown, its involve other proteins, like calmodulin-dependent protein kinase II (CAMK-II), cyclin, and cohesin, which hold the chromosomes together, and the inactivation of the mitogen-activated protein (MAP)-kinase, involve in the increase of DNA synthesis. All these are critical for the downstream signaling cascade modulated by Ca^+ 2^ release [2, 18, 19]. It has been demonstrated that the specific frequency of Ca^+ 2^ spikes can affect oocyte activation and early embryo development, resulting in fewer pregnancies [20]. The reason for this is the need for two constant and simultaneous active stimuli: cyclin B synthesis and cyclin-dependent kinase 1 (CDK1). Cyclin B maintains CDK1 activity, while CDK1 holds the oocyte in meiotic arrest. A spike in intracellular Ca^+ 2^ levels increases cyclin B’s proteolysis, reducing CDK1 activity and causing the resumption of oocyte meiosis [21]. However, if only one spike in intracellular Ca^+ 2^ levels occurs, cyclin B restores its synthesis, CDK1 increases its activity, and the oocyte is re-arrested [21, 22].

The secretions of metabolic enzymes, gene expressions, among other molecular and cellular functions, are related to the intracellular Ca^+ 2^ rise. So, Ca^+ 2^ plays the second messenger role and is responsible for releasing the oocytes from the meiotic arrest and triggered the *embryonic development program* [23].

Different theories involving intracellular Ca^+ 2^ oscillations during fertilization have been introduced, including the injection of Ca^+ 2^ in the ooplasm by a sperm membrane Ca^+ 2^ bomb or channel [24] and the sperm oocyte interaction hypothesis [25], in which an interaction between a sperm ligand and an oocyte receptor triggers Ca^+ 2^ oscillation. Although the exact mechanism of intracellular Ca^+ 2^ release is not clear, a consensus for SOAF triggering the release of intracellular Ca^+ 2^ upon fertilization exists, as well as the participation of other agents, such as cyclic GMP (cGMP), cyclic ADP-ribose (cADP ribose), nicotinic acid adenine dinucleotide phosphate (NADP) and nitric oxide (NO) in the rise of intracellular Ca^+ 2^ [19].

## Sperm oocyte-activating factors

The development of new biotechnological tools that can be applied in diagnosis and clinical research, such as Ca^+ 2^ imaging and ICSI, has led to the dismissal of the hypotheses of the Ca^+ 2^ bomb or ligand-receptor mechanisms. Although both theories explain intracellular Ca^+ 2^ increase, both failed in explaining how Ca^+ 2^ oscillations persist over time [20, 26, 27]. In contrast, the “sperm factor” theory, according to which oocyte activation is triggered by a soluble sperm factor released into the oocyte, was rapidly accepted.

**Fig. 3:**
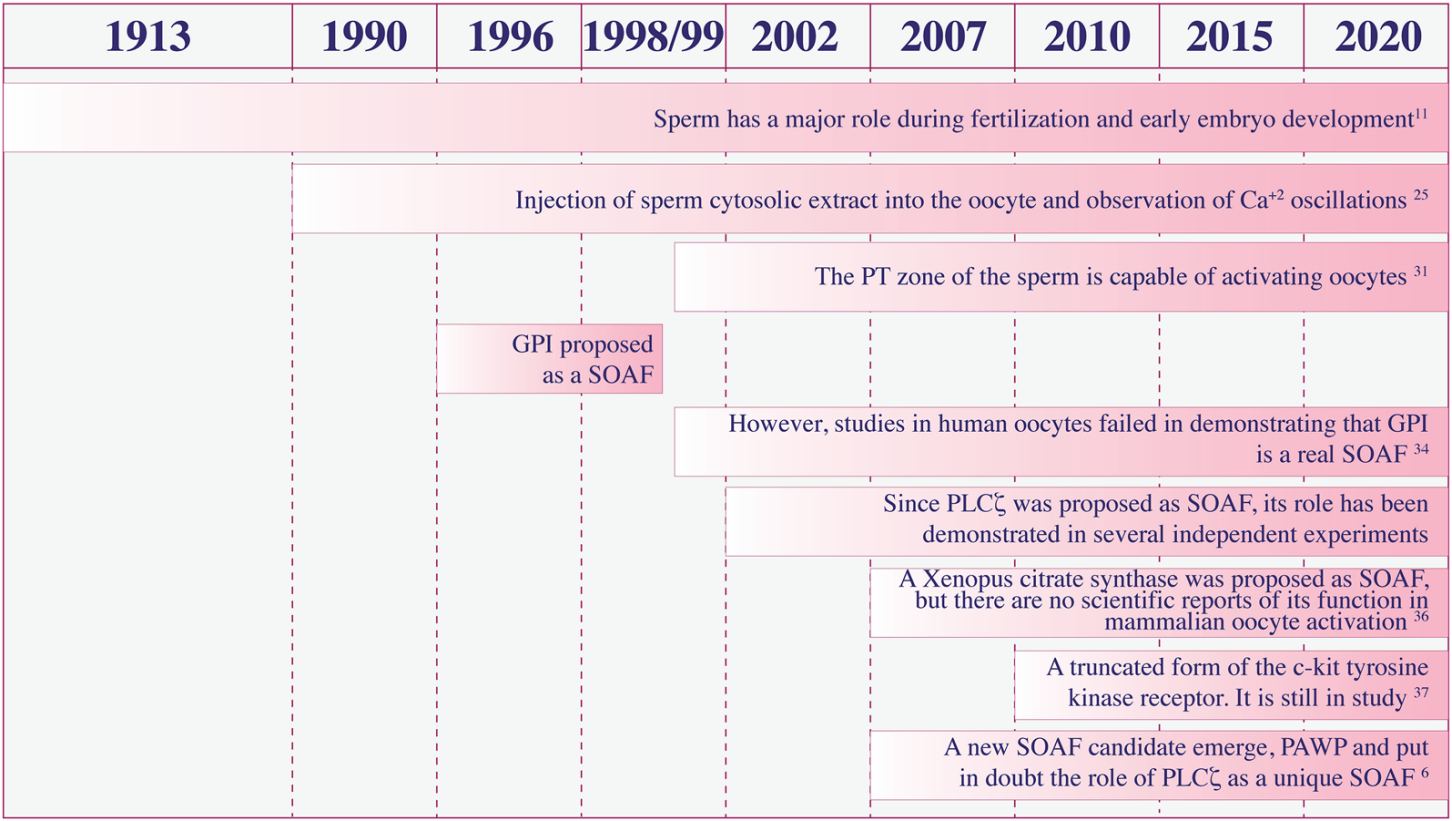
A History of SOAF candidates. A timeline with the most important reports about the role of the sperm factor candidates in the oocyte activation mechanism

Since Loeb´s principle in 1913, evidence about sperm ability to activate oocyte has been increasing and has been proposed several “sperm factors” (Fig. 3). Swann et al. provided the first reported evidence for this sperm factor [28] by injecting a sperm cytosolic extract into the oocyte and observing Ca^+ 2^ oscillations, like those observed during fertilization. This factor remained unidentified for several years before indirect evidence in literature helped define it.

The SOAF must possess the following specific characteristics and functions: it should be capable of triggering persistent oscillations of intracellular Ca^+ 2^ from ooplasmic resources in a manner indistinguishable from mammalian fertilization. This mechanism should involve the increased production of IP_3_, regulated by the phosphoinositide-signaling pathway.

Location was also important for the SOAF function (Fig. 2). The sperm head has a region called the perinuclear theca (PT), a condensed cytosolic protein layer that surrounds the nucleus and can be divided into structural or functional zones [29]. The functional zones are divided into three further parts: a subacrosomal region, equatorial segment, and postacrosomal sheath-perinuclear theca (PAS-PT). The PT contains proteins that maintain the sperm head’s structure and coat the nucleus (Fig. 2). When fertilization begins, the sperm’s fusion with the oocyte begins in the equatorial segment and continues to the PAS-PT, thus this last region being the first that is exposed to the ooplasm. Experiments assessing the regions of the sperm capable of activating oocytes, involved an injection of the head or tail of a spermatozoon into mouse oocytes [30], indicating that only the sperm head can activate the oocyte. Moreover, when sperm heads were treated with substances that alter all membranes, such as proteases or detergents that denaturalize proteins, sperm failed to activate the oocytes. Meanwhile, when treated with Triton X-100, a non-ionic surfactant that removes all membranes except the PT around the nucleus, the sperm retains the ability for activation [31]. Data show that SOAF could be a protein [28, 32]; however, most of the proteins investigated did not match the physiological characteristics expected of an oocyte activator factor.

The first candidate was a glucosamine-6-phosphate isomerase (GPI), a deaminase homologue to the hamster oscillin, and a putative soluble protein associated with Ca^+ 2^ oscillation-inducing activity in mammalian oocytes, located in the equatorial segment of the sperm head [33]. However, injection of hamster oscillin did not induce Ca^+ 2^ oscillations in mouse oocytes [34]. Likewise, a recombinant form of the human GPI also did not induce Ca^+ 2^ oscillations [35]. Immunodepletion of GPI from sperm extracts failed to block Ca^+ 2^ oscillations [35].

Another SOAF candidate considered was a homolog of *Xenopus* citrate synthase. This 45-kDa protein was controversial; it triggered Ca^+ 2^ oscillations in unfertilized newt oocytes, and treatment with anti-citrate synthase antibody in sperm extracts reduced oocyte activation. There are no scientific reports of its function in mammalian oocyte activation or in the fertilization process [36].

A truncated version of the c-kit tyrosine kinase receptor, Tr-Kit, was another SOAF candidate. Although this receptor’s role in mammalian fertilization is still unknown, it is expressed in the equatorial region of the sperm head and persists after the acrosome reaction in high-quality sperm [37, 38].

Sperm extract that triggers Ca^+ 2^ oscillations contains higher PLC activity, and this stimulation can be differentiated from oocyte PLC activity [39]. As the PLC family of proteins has a critical function in the oocyte activation cascade, they became a major focus of clinical research [14, 40]. These proteins catalyze the hydrolysis of PIP_2_, generating IP_3_ and DAG, allowing IP_3_ to bind its receptor and release intracellular Ca^+ 2^ resources from the ER. This rise of intracellular Ca^+ 2^ levels activates the protein kinase C pathway, and this signal is decoded into a cellular response [28, 41, 42].

This family of PLCs currently has 13 isozymes [43]. Although multiple isoforms of this family are only expressed in testis and sperm [44], the evidence for SOAF points to PLCζ. Discovered in 2002, this novel, sperm-specific PLC is the smallest of the PLC family [45, 46] and sensitive to environmental Ca^+ 2^ concentrations, unlike other PLCs [47, 48]. The dominant role of this protein has been demonstrated in several independent experiments, corroborating a series of features that made PLCζ a great SOAF candidate, in addition to its presence in the sperm head. Injection of PLCζ mRNA into mouse oocytes induced a fertilization-like Ca^+ 2^ oscillation profile [45, 46], while other isoforms, like PLCγ mRNA, failed to do the same. Likewise, the depletion of PLCζ in porcine or hamster sperm extracts had a reduced ability to initiate Ca^+ 2^ oscillations in oocytes [46]. Corroborating this, a transgenic mouse model, created via RNA interference technology, with PLCζ-deficient sperm did not produce Ca^+ 2^ oscillations after sperm injection [49]. PLCζ was found to be located in the equatorial segment and postacrosomal region by immunofluorescence and electron microscopy [9, 50, 51], associated with the inner acrosomal membrane [52]. Human sperm contains variable amounts of PLCζ, that is why it possesses a variable ability to cause Ca^+ 2^ oscillations [3, 53]. The expression patterns of this protein may play an important role in Ca^+ 2^ oscillations, and these functional roles might not be limited to oocyte activation [54, 55]. The association between PLCζ activity and male infertility has also been demonstrated by the expression of PLCζ in human sperm heads [56].

**Fig. 4:**
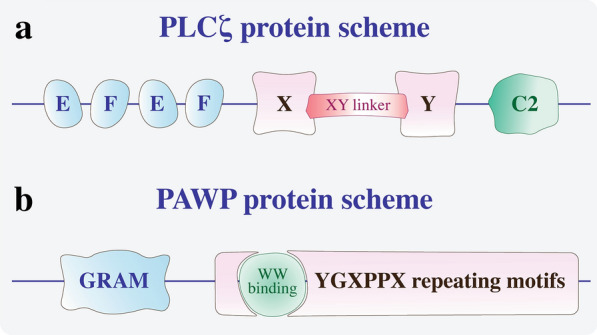
SOAFs protein scheme. **a** PLCζ protein scheme showing two pairs of EF domains involved in Ca^+ 2^ binding, a XY domain associated with catalytic activity with a XY-linker (a putative NLS species-specific), and a C2 domain involved in IP_3_ binding. **b** PAWP protein scheme with the WW binding region of the YGXPPX repeating motifs and the N-terminal GRAM domain

The structure and functional domains of the SOAF may play an important role [57]. The PLCζ structure consists of four EF-hand domains, a C2 domain, and a catalytic X and Y core domain. The main characteristics that differentiate PLCζ from the other PLC isoforms are the EF3 zone, responsible for high Ca^+ 2^ sensitivity and the lack of a PH domain, related to the capacity of linked to G-proteins in the membranes and reacts with PIP_2_ [57, 58] (Fig. 4a).

Despite the evidence, studies using two different knockout mouse models for PLCζ show that male sperm can produce offspring with reduced litter size [59, 60]. These PLCζ-deficient sperm can fertilize oocytes, inducing a reduced but sufficient number of Ca^+ 2^ oscillations. The number of oscillations rises to 70% in oocytes activated by delayed pronuclei formation and increased polyspermy [60]. These results show that there is likely another unknown SOAF that can activate oocytes in the absence of PLCζ. This unknown SOAF can activate oocytes when fresh sperm fertilizes oocytes in IVF, but not with the ICSI technique [61]. Nevertheless, PLCζ is the main SOAF for stimulating oocyte activation in mammals. The unknown SOAF could compensate for some pathologies of PLCζ deficiency and could be a new target for pharmaceutical agents, requiring further investigation. However, this unknown SOAF in the human sperm has not yet been confirmed [61, 62].

However, a SOAF candidate emerged as an alternative to PLCζ: postacrosomal WW domain-binding protein (PAWP), a WBP2 N-terminal like protein [6, 63, 64]. PAWP, an alkaline protein with an N-terminal WW binding domain and a C-terminal domain with a PPXY consensus binding site for group-1 WW domain-containing proteins [6, 63]. It is located in the perinuclear matrix of the sperm head (Fig. 4b). In 2007, Wu et al. reported that PAWP promotes meiotic resumption and initiates pronuclear development during fertilization in bovine, pig, monkey, and *Xenopus* oocytes [6]. This finding was confirmed several years later [63] when a recombinant PAWP was injected into MII-oocytes and triggered an increase in intracellular Ca^+ 2^ levels. However, the injection of a competitive inhibitor of this protein prevented the release of intracellular Ca^+ 2^ and oscillations [65]. Other independent groups have demonstrated the association between PAWP expression and the competence of human and bull sperm [65, 66]. However, some authors still cannot confirm a relationship between SOAF expression and Ca^+ 2^ oscillations [59, 67]. In addition, sperm from PAWP knockout mice induced normal Ca^+ 2^ oscillations in oocytes after IVF and ICSI [68].

Despite the data showing that PAWP fulfilled all the relevant requirements to be classified as a SOAF, the mechanism of PAWP inducing Ca^+ 2^ release from the ER is merely theoretical. Some authors proposed that the effects of PAWP are mediated via other proteins, like the yes-associated protein, which activates PLCγ and, subsequently, the IP_3_ signaling pathway downstream [6, 65]. Mehlmann et al. (1998) used an exogenous growth-factor expression to stimulate the PLCγ pathway, allowing Ca^+ 2^ oscillations in mouse oocytes [27]. However, the relationship between PAWP expression, PLCγ activity, and Ca^+ 2^ oscillations could not be demonstrated; plus, the rise of intracellular Ca^+ 2^ levels mediated by PAWP did not show wavelike characteristics, the trademark of mammalian fertilization [27, 67]. Conversely, no conclusive evidence shows that this PLC isozyme, PLCγ, plays a significant role in oocyte activation [27], and is not as sensitive to Ca^+ 2^ as PLCζ [47].

At present, the complete molecular mechanism by which SOAF, PLCζ or PAWP, activates the oocyte still remains unclear. Moreover, one of the theories proposes that PAWP activates PLCζ, and then PLCζ hydrolyzes PIP_2_ [58]. However, this mechanism requires further research, Aarabi et al. (2014) suggest that the C-terminal domain of PAWP, which is rich in proline and presents a PPXY consensus sequence, interacts with a WW-group I protein domain from PLCζ, and activate it. This activation of PLCζ allows the hydrolyzation of PIP_2_ and trigger the Ca^+ 2^ oscillations. The authors propose that when the PPXY region of PAWP is blocked, the oocyte activation failed [65].

## Epigenetic regulation during fertilization

The sperm epigenome modifications include DNA methylation, post-transcriptional modifications of histones, and non-coding RNAs (ncRNAs) [69]. These epigenetic events affect early embryo development, and cause phenotypic changes in the offspring [70]. During fertilization, sperm DNA compaction and protamine replacement are the most important events to form the zygote nucleus, while ncRNAs are involved in embryonic development and transgenerational adaptation [69].

Sperm chromatin includes testicular histones, protamines, and free DNA, called nuclear matrix. The complex transition between the oocyte and the spermatozoon chromatin structure is poorly understood, but it is known that in the spermatozoon, the protamine incorporation during spermatogenesis, as well as its removal upon fecundation are, apparently, critical for paternal epigenetics profile and reprogramming. It has been demonstrated that there is non-random retention of testicular histones in a specific region of the DNA during spermatogenesis, and the more “relaxed” state of the DNA in these regions provides conditions to epigenetic marks [70–72]. In contrast to the passive demethylation that occurs in the oocyte DNA upon fertilization, the sperm chromatin undergoes throughout deprotamination followed by dramatic demethylation and active decondesation of the chromatin, creating an environment that facilitates a new methylation profile, thus new epigenetics marks, suggesting that determination of cellular fates for tissue specification is critical [73–76].

ncRNAs are involved in gene expression either by cleavage targets mRNAs or by blocking mRNAs translation. Although the presence of different types of ncRNAs in the sperm has been demonstrated, the function of these during fertilization is not clear [69].

## Oocyte activation failures

After fusion of the sperm with the oocyte membrane, pronucleus formation occurs. Lack of pronuclei is a clear sign of failed fertilization during ICSI. The mechanism through which the human oocyte is activated during ICSI is different from natural fertilization, yet approximately 85% of attempts successfully lead to pregnancy. However, a percentage of ICSI cycle failure has been reported [77], even with good ovarian response and semen quality. As the spermatozoon is already within the oocyte, the fertilization failure could be associated with sperm factors or oocyte activation machinery. Failed fertilization after ICSI may be attributable to the lack of Ca^+ 2^ oscillations, both related to total or partial oocyte fertilization [61].

TFF occurs when all the oocytes collected within one cycle of stimulation fail to form pronuclei after ICSI; OAD is the main cause of TFF in recurrent ICSI failure [78–80]. Between 1 and 5% of ICSI cycles repeatedly fail, associated with abortive oocyte activation [8, 81–83]. Numerous reports have shown that sperm factors affect oocyte activation, specifically the lack of PLCζ or mutation in PLCζ in the sperm [50, 84]. The absence of the PLCζ protein in the sperm head is directly associated with the failure to produce Ca^+ 2^ oscillations [8, 84]; therefore, an effective way to determine the cause of ICSI failure is to inject human sperm into mouse oocytes and observe the spermatozoon’s ability to trigger Ca^+ 2^ oscillations. Other causes of OAD are mutations in the PLCζ gene that inhibit PLCζ enzymatic activity [50]. A notable example is the case of two brothers with homozygous mutations in the C domain of PLCζ; this mutation reduced the PLCζ expression in the sperm and disrupted activity [85]. These findings strongly suggest that this mutation can cause male infertility in humans.

Another genetic pathology known as globozoospermia is associated with the capability of activating oocytes. This autosomal-recessive pathology, which can be partial or total, is commonly caused by mutations in the DPY19L2 gene involved in developing the acrosome and elongation of the sperm head; therefore, the spermatozoon lacks the acrosome or shows an abnormality in other structures that provide its characteristic shape [8, 50]. Many cases of globozoospermia are associated with a lack of PLCζ [52]. Less common causes of TFF are sperm head decondensation, premature sperm chromatin condensation, oocyte spindle defects, and sperm defects [86].

The type of failed fertilization related to oocyte activation after an ICSI cycle is associated with the low ability of the sperm to stimulate Ca^+ 2^ oscillations [61]. Ferrer-Buitrago et al. (2018) found that 30% of control sperm from fertile men could not effectively prolong Ca^+ 2^ signaling in human oocytes [87]. This report is consistent with the immunostaining results of sperm from fertile men, which showed a variable localization and amount of PLCζ [3].

## Current situation of diagnosis and treatment for oocyte activation failure

ARTs involve ovarian stimulation, gamete and embryo manipulation, and cryopreservation. At present, IVF and ICSI are reproductive technologies that are widely used to treat infertility related to reproductive endocrinology, genetic disorders, oocyte donation, and surrogacy. The aim of ART is to attain a successful pregnancy, and during this process, most biological barriers are bypassed, especially when ICSI is applied, because a morphologically normal spermatozoon is directly injected into a mature oocyte [7]. Since the early 1990s, when the first pregnancy using ICSI was reported [88], almost any type of spermatozoa were used to fertilize an oocyte. Today, the clinical situation has improved with research, allowing the investigation and determination of gene expression, proteins, and molecular pathways related to gamete formation and development, fertilization processes, and embryo development.

The lack of pronuclei formation after a conventional IVF procedure is a clear sign of fertilization failure. The reasons can be multiple, such as non-recognition between the spermatozoon and the oocyte or failure of acrosome reaction, among others. Currently, ICSI is the preferred treatment when conventional IVF fails. Applied, for example, when oocytes are cryopreserved or in severe male infertility cases. Although there is controversy, there are two main sources of potentially usable sperm, testicular or epididymal spermatozoa obtained through biopsies or ejaculated sperm. While the protamine content in the ejaculated spermatozoa confers the ability to be reprogrammed after fertilization [89], thus the ejaculated sperm is thought to be more mature; it has been proposed that the extraction of testicular sperm may eliminate the exposure to the reactive oxygen species and could result in the access to high-quality spermatozoa [90].

According to the Human Fertilization and Embryology Authority, the leading cause for ICSI treatment in about 50% of the cases [91] is related to the male factor. However, most cases are successful, but there is a small percentage of cases in which conventional ICSI fails. The mechanisms that are altered are unknown, and researchers are currently uncertain of solutions. In most cases, other complementary strategies, such as chemical adjuvants or alternative techniques, are applied to improve fertilization and achieve better embryo development.

As previously discussed, OAD is the most common cause of ICSI cycle failure. Oocyte activation is a complex and not completely known sequence of molecular events that includes gamete membrane fusion, exocytosis of cortical granules of the oocyte, oocyte intracellular Ca^+ 2^ release and oscillations, recruitment of maternal mRNA, pronucleus formation, and polyspermy prevention. Localization patterns of proteins, receptors, DNA, and membrane integrity help elucidate the stages in oocyte activation and reasons for failures; they also help to develop diagnostic tests and therapeutic strategies to restore fertilization, either through pharmaceutical agents or reproductive technology or both.

Increasing evidence shows that PLCζ is the most important SOAF. It activates Ca^+ 2^ oscillations through PIP_2_ hydrolysis to IP_3_ and DAG. On the ER’s surface, IP_3_ binds to its receptors and triggers Ca^+ 2^ release in a wave pattern. As previously demonstrated, OAD could be caused by abnormal patterns of distribution or reduced amounts of PLCζ, among other factors, which could lead to a failure in the release and oscillations of Ca^+ 2^ [3, 84, 92], altering the downstream pathway. In reproductive medicine, when Ca^+ 2^ oscillations fail, mainly because of the absence or diminished amount of PLCζ, the most common procedure is artificial activation of the oocytes prior to ICSI by applying AOAs (Fig. 1—artificial activation).

Artificial stimulation of oocyte activation through Ca^+ 2^ ionophores in different animals has been studied since the 1970s. Initial studies showed that the ionophore A23187 released Ca^+ 2^ from intracellular stores, and the direct injection of Ca^+ 2^ into mouse oocytes triggered the induction of parthenogenetic embryos that developed to the blastocyst stage [18]. While some authors demonstrated that human oocytes could also be activated by A23187, other groups found that A23187 and ionomycin, another ionophore similar to A23187, only cause a unique spike in Ca^+ 2^ and do not activate oocytes [93, 94]. The AOAs do not mimic the fertilization process precisely but cause a single large Ca^+ 2^ spike, which is not the natural Ca^+ 2^ oscillations [18, 94, 95]. Therefore, to activate oocytes, the most common protocol applied in humans includes the ready-to-use ionophore A23187, following ICSI [18, 96].

AOAs have been used for over a decade in reproductive medicine. Many reports have shown the beneficial effects of AOAs in reproductive medicine in couples with TFF [18, 97], male patients with severe sperm alterations including globozoospermia [98, 99], teratozoospermia [80, 100], cryptozoospermia, azoospermia [101], and sperm stress conditions, such as cryopreservation protocols [102, 103]. In 2017, Murugesu et al. reported that using a Ca^+ 2^ ionophore in ICSI treatments significantly improved oocyte activation and pregnancy rates [104]. Reports using AOAs in an ICSI cycle are contradictory, so it is possible that only a subset of patients will benefit from them [105].

Concern still exists regarding the potentially deleterious effects of these substances on embryogenesis [106]. Vanden Meerschaut et al. conducted a study on neonatal and neurodevelopmental outcomes in 21 children born after an ICSI-AOA treatment [107]. This group reported no severe effects in the offspring. However, the high response rate and the robustness of the test used in this study are still considered preliminary because the sample size was small.

An alternative treatment for OAD is microinjections of PLCζ protein or as a recombinant protein (Fig. 1—artificial activation). It has been demonstrated that both recombinant PLCζ and PLCζ RNA trigger intracellular Ca^+ 2^ oscillations in both mouse and human oocytes [9, 103, 108–110]. The technical problem of PLCζ RNA injections is the variable expression between oocytes, it is known that exist species-specific Ca^+ 2^ oscillatory patters, and the type of patter affects preimplantation embryonic development [14]. However, the exact mechanism is still unclear; in some species, such as mice or pigs, the Ca^+ 2^ oscillatory pattern dependent on the nuclear localization signal sequence of PLCζ [111]. Thus, injection of PLCζ may be useful for the activation of round spermatid-injected and somatic nuclear-transferred oocytes, but the overexpression could lead to the cleavage-stage arrest of the oocyte [14, 111, 112], so this treatment is problematic and difficult to apply in reproductive medicine. Another important problem is the introduction of genetic material into the oocyte, which is forbidden for human medicine in most parts of the world. In contrast, recombinant PLCζ could by synthesized in bacteria as a fusion protein. This resolves the problem of varying PLCζ expression but gives rise to PLCζ diminishing its activity quickly. Therefore, recombinant PLCζ protein must be stabilized and calibrated before its application [61]. Still, its application in IVF clinics is limited because of commercial availability.

As an alternative, there are treatments with external agents, including pharmacological, chemical, or microinjection with PLCζ. Authors have proposed that routine sperm preparation methods, including density gradient selection or swim-up (selecting sperm by motility and morphology), be modified and include other selected molecular or cellular sperm characteristics. One example is selecting for surface markers using magnetically activated cell sorting (MACS) for the selection of apoptotic sperm, which express phosphatidylserine in their membrane [113]. These modifications of preparation methods could improve sperm selection and improve fertilization treatments. Thus, Chan et al. suggested a method based on zeta-potential selection according to the electric charge that could produce a higher percentage of normal sperm morphology with intact chromatin [114]. In accordance, Khakpour et al. suggested a noninvasive method based on zeta potential, along with the density-gradient selection method, which improved the intact chromatin and membrane selection of a morphologically normal spermatozoon, with a high amount of PLCζ, important characteristics required for fertilization and oocyte activation [77].

Another strategy, proposed by authors, is a simple protocol involving the incubation of the oocytes for several minutes in media containing Strontium (Sr^+ 2^) [115]. Although this strategy produces Ca^+ 2^ oscillation in mouse [116] and bovine [117] oocytes, in reproductive medicine, this protocol has “anecdotal clinical reports” without correct scientific methodology [61]. It has been demonstrated the presence and functionality of these channels in human oocytes, the efficiency, and the exact mechanism of Sr^2+^ as an oocyte activation agent in human oocytes remains largely unknown [118]. Many authors have tried to corroborate this finding in mouse oocytes but failed, with over 10 hours of incubation in Sr^+ 2^ media [61, 87, 118]. This lack of response was also observed in cows and pigs [61, 116]. However, Norozi-Hafshejani et al. obtained quality embryos derived from SrCl_2_ activation prior to ICSI, although this treatment was less efficient than the Ca-ionophore stimulus [115].

These strategies enable solutions for infertility problems related to the spermatozoon, such as globozoospermia or recurrent ICSI cycle failure because of OAD. It must be noted that not all cases are similar, so treatment could differ among patients, and some recommendations/guidelines must be followed. The AOA treatments are only recommended in cases when PLCζ deficiency has been observed [119]. However, when sperm preparation procedures target some characteristics of the spermatozoon, such as DNA fragmentation levels or acrosome and membrane integrity, modifications of the preparation method must be chosen with care [120].

## Discussion

Since Jacques Loeb proposed that the spermatozoon plays a key role in fertilization, more than just providing genetic material [11], a new field in reproductive biology opened a series of investigations and debates on the role of the male gamete in early embryo development and its effects on the offspring. This resulted in the knowledge of spermatozoon factors as fundamental to initiating oocyte activation, and the sperm epigenome important role for successful embryogenesis.

Numerous hypotheses have been offered explaining how the sperm activates the oocyte by increasing intracellular Ca^+ 2^ oscillations. Many factors and molecular pathways have been studied to determine pronuclear formation, for which the oocyte machinery modifies the sperm chromatin structure after fertilization.

Several mammals, as well as heterologous ICSI, have been used as model systems to study oocyte activation. Among these, similar observations between human and mouse oocyte fertilization and activation mechanism and embryo development processes allow us to compare and contrast the details [9, 84, 92, 112, 121–124]. The development of ART, especially ICSI techniques that bypass any oocyte-sperm membrane-binding mechanism, concluded with a soluble SOAF that enters the oocyte upon fertilization, surviving to the acrosome reaction.

The discovery of PLCζ and the demonstrations of its role as a SOAF is vital [14], plus another potential candidate is under investigation, PAWP (Table 1). However, while both meet the requirements proposed, sperm location and ability to trigger Ca^+ 2^ oscillations signals, at present, do not explain how PAWP triggers intracellular Ca^+ 2^ release; therefore, its mechanism remains theoretical [125].

**Table 1:** Descriptions of PLCζ and PAWP and its implication in ART

Factor	Description	Biological function	Molecular functions	Alteration in OAD	References
PLCζ	The smallest sperm-specific PLC, consist in four EF hand domains, a C2 domains, and a catalytic X and Y core domain, located in the equatorial segment and PAS-PT region. Sensitive to environmental Ca^+ 2^ concentrations	Triggers intracellular Ca^2+^oscillations in oocytes during M phase and is involved in inducing oocyte activation via IP_3_. May exert an inhibitory effect on PLC-coupled processes that depend on Ca^+ 2^ and PKC, including CFTR trafficking and function	Ca^+ 2^ binding, IP_3_ binding, PIP_2_ binding, phosphatidylinositol-5-phosphate binding, phospholipase activity	Deficiencies, abnormal localization, activity/expression, or genetic mutations in PLCζ have been linked ICSI failure or infertile men Point mutations in the PLCζ gene have been identified a globozoospermic and non-globozoospermic infertile patient resulting in a deficiency of oocytes activation	[2, 10, 17–19, 43, 44, 48–50]
PAWP	A PAS-PT WW domain-binding protein located in the post-acrosomal sheath of the sperm	May play a role in meiotic resumption and pronuclear formation during fertilization	Chromatin DNA binding, transcription coactivator activity, WW domain binding	In has been associated, via immunohistochemistry, the level of PAWP and sperm quality and fertilizing ability It has been proposed that when the PPXY region of PAWP is blocked, the oocyte activation failed	[7, 58–60]

Since all IVF clinics do not have access to animals for assays, particularly mouse models to evaluate heterologous ICSI and the ability of the human sperm to activate mouse oocytes, a standardized protocol should be developed. This protocol must have all necessary biochemical characteristics, including analytical and biological limits, a scientific validation, reference values or range, normal variation, and a consensus of the professional community when alterations are observed [126]. Evaluating PLCζ and PAWP expression in infertile men with previous fertilization failure by immunostaining showed that both proteins were under expressed in patient sperm. However, the authors proposed that both proteins are SOAF candidates and could be diagnostic markers; the findings are ambiguous since a lack of common diagnosis protocols can determine PLCζ activity or PAWP. Thus, treatments are empirical since not all patients respond to AOA treatments and each IVF clinic has its own protocol for such cases.

Alterations in gamete development or the fertilization process show that embryogenesis may fail or that multiple disorders could appear in offspring. In human reproduction, maternal, paternal, or idiopathic factors could cause this. Therefore, new advances in biotechnology have informed reproductive medicine to manage these pathologies. The use of ARTs in human infertility cases, such as ICSI and preimplantation genetic diagnosis or screening (PGD or PGS), has been enormously useful for morphologically and genetically normal embryo transfer, plus the study of fertility failures. In the future, additional factors or mechanisms associated with oocyte maturation must be studied. It would be misguided to assume that only a single sperm factor activates oocytes. In the same way, other functions of these SOAF must be studied; for example, there is evidence that PLCζ has a potential role in embryonic development, particularly during the early embryonic division [20, 127].

The role and importance of both sperm factors, PLCζ and PAWP, among others oocytes factors and proteins, such as CAMK-II or other protein kinases, must be continuously studied to expand our knowledge and improve diagnosis and clinical treatments for infertility.

## Conclusions

Through those years, the role of the spermatozoon during fertilization and beyond has become important. It is known that the sperm has an inactive role during oocyte activation, dues several sperm factors were associated with this. However, PLCζ has been demonstrated to be the main SOAF; with an uncountable number of scientific data, it seems no to be the only one. In recent years, other candidates have risen, and this is the case of PAWP. Although the mechanism by which PAWP could activate the oocytes is not clear, many authors defend it as a SOAF. Many biological pathways are ambiguous; this may represent an alternative in the case that the main pathway is altered.

It is worth pointing out that SOAF identification and mechanism were important to study and treat male infertility, such as male cause of OAD. Nowadays, it is known that the deficiency of PLCζ in a sperm sample could lead to a fertilization failure. However, no diagnostic test has been developed to establish the amount of PLCζ, as well as a standard protocol to deal with this type of pathologies.

## Data Availability

Not applicable.

## Abbreviations

AOAs: Artificial oocyte activators
AOD: Oocyte activation deficiency
ART: Assisted reproductive technology
Ca^+ 2^: Intracellular calcium
cADP ribose: Cyclic ADP-ribose
CAMK-II: Calmodulin-dependent protein kinase II
CDK-1: Cyclin-dependent kinase 1
CFTR: Cystic fibrosis transmembrane conductance regulator
cGMP: Cyclic GMP
CIRC: Ca^+ 2^-induced Ca^+ 2^ release
DAG: Diacylglycerol
DNA: Deoxyribonucleic acid
ER: Endoplasmic reticulum
GPI: Glucosamine-6-phosphate isomerase
ICSI: Intracytoplasmic sperm injection
IVF: In vitro fertilization
IP_3_: Inositol-1,4,5- triphosphate
IP_3_R: Inositol-1,4,5- triphosphate receptors
MACS: Magnetically activated cell sorting
MAP-kinase: Mitogen-activated protein kinase
NADP: Nicotinic acid adenine dinucleotide phosphate
ncRNAs: Non-coding RNAs
NLS: Nuclear localization signal
NO: Nitric oxide
OAD: Oocyte activation deficiency
PAS-PT: Postacrosomal sheath-perinuclear theca
PAWP: Postacrosomal WW domain-binding protein
PGD: Preimplantation genetic diagnosis
PGS: Preimplantation genetic screening
PIP_2_: Phosphatidylinositol 4,5-biphosphate
PKC: Protein kinase C
PLC: Phospholipase
PLCγ: Phospholipase gamma
PLCζ: Phospholipase C zeta 1
PT: Perinuclear theca
RNA: Ribonucleic acid
RYR: Ryanodine receptors
SOAF: Sperm oocyte-activator factor
Sr^+ 2^: Strontium
TFF: Total fertilization failure
Tr-Kit: c-kit tyrosine kinase
